# Impact of preterm birth on infant mortality for newborns with congenital heart defects: The EPICARD population-based cohort study

**DOI:** 10.1186/s12887-017-0875-z

**Published:** 2017-05-15

**Authors:** Enora Laas, Nathalie Lelong, Pierre-Yves Ancel, Damien Bonnet, Lucile Houyel, Jean-François Magny, Thibaut Andrieu, François Goffinet, Babak Khoshnood, on behalf of the EPICARD study group, François Goffinet, Babak Khoshnood, Damien Bonnet, Drina Candilis, Anne-Lise Delezoide, Lucile Houyel, Jean-Marie Jouannic, Nathalie Lelong, Suzel Magnier, Jean-François Magny, Caroline Rambaud, Dominique Salomon, Johanna Calderon, Thibaut Andrieu, Anne-Claire Thieulin, Véronique Vodovar, Maggy Chausson, Anissa Brinis, Laure Faure, Maryline Delattre, Jean-Marc Treluyer, Gérard Bréart, Dominique Cabrol, Alain Sérraf, Daniel Sidi, Marcel Voyer

**Affiliations:** 1 Obstetrical, perinatal and pediatric epidemiology research team, Center for biostatistics and epidemiology, INSERM U1153, Maternité de Port-Royal, 6ème étage, 53 av. de l’Observatoire, 75014 Paris, France; 2 Service de chirurgie des cardiopathies congénitales, Hôpital Marie Lannelongue, 133, avenue de la Résistance, 92350 Le Plessis Robinson, France; 3 Centre de référence M3C-Necker, Université Paris Descartes, 140 rue de Sèvres, 75015 Paris, France; 4 Service de pédiatrie et réanimation néonatales CHU Necker Enfants Malades, Paris, France; 5 Maternité Port Royal, 53 avenue de l’Observatoire, 75014 Paris, France

**Keywords:** Preterm birth, Congenital heart defects, Mortality

## Abstract

**Background:**

Congenital heart defects (CHD) and preterm birth (PTB) are major causes of infant mortality. However, limited data exist on risk of mortality associated with PTB for newborns with CHD. Our objective was to assess impact of PTB on risk of infant mortality for newborns with CHD, while taking into account the role of associated anomalies and other potentially confounding factors.

**Methods:**

We used data on 2172 live births from a prospective population-based cohort study of CHD (the EPICARD Study) and compared neonatal, post-neonatal and overall infant mortality for infants born at <32, 32–34 and 35–36 weeks vs. those born at term (37–41 weeks).

**Results:**

Preterm newborns had a 3.8-fold higher risk of infant death (17.9%) than term newborns (4.7%), RR 3.8, 95%CI 2.7–5.2; the risk associated with PTB was more than four-fold higher for neonatal (RR 4.3, 95% CI 2.9–6.6) and three-fold higher for post-neonatal deaths (RR 3.0, 95% CI 1.7–5.2). Survival analysis showed that newborns <35 weeks had a higher risk of mortality, which decreased but persisted after exclusion of associated anomalies and adjustment for potential confounders.

**Conclusions:**

Preterm birth is associated with an approximately four-fold higher risk of infant mortality for newborns with CHD. This excess risk appears to be mostly limited to newborns <35 weeks of gestation and is disproportionately due to early deaths.

## Background

Preterm birth (PTB) and congenital heart defects (CHD) are two major causes of mortality, morbidity and disability of perinatal origin [1–4]. Moreover, newborns with CHD are at a higher risk of PTB [5, 6] and both PTB and low birth weight have been shown to be risk factors for hospital mortality for newborns with CHD [7–10].

Reported infant mortality rates for newborns with CHD and preterm birth range from 20% [5] to 65% [11] with most of the deaths occurring during the first 28 days of life. There is some evidence suggesting that progress in medical and surgical management of newborns with CHD, and/or specifically those with PTB, has resulted in more favorable outcomes for infants undergoing cardiac surgery [12, 13]. However, by far most of the available literature looking at the impact of PTB on the outcomes of CHD are based on hospital series [7, 14–17] and concern essentially cases of cardiac surgery in specialized centers.

In the only previous population-based study reporting on the impact of PTB on the risk of infant mortality for newborns with CHD [5], the main focus was on the relation between risk of PTB and presence of CHD. The authors also reported descriptive data on the risk of infant mortality for CHD associated with PTB. They found that the overall infant mortality rate for newborns with CHD was 13%, whereas for the subgroup of preterm infants with CHD, risk of mortality was 20%. This study did not examine the role of associated anomalies, the degree of severity of CHD and the effects of potentially confounding factors, notably multiple pregnancies and intrauterine growth restriction (IUGR) on the risk of mortality associated with preterm birth for newborns with CHD.

Using data from a population-based cohort study of infants with CHD (the EPICARD study), we examined the association between PTB and the risk of infant mortality for newborns with CHD for: i) all CHD combined, ii) “isolated” CHD (excluding CHD associated with chromosomal or other anomalies) and iii) “isolated major CHD” (isolated CHD excluding ventricular septal defects).

## Methods

### Data source

EPICARD (Etude EPIdémiologique sur le devenir des enfants porteurs de CARDiopathies congénitales) is an ongoing prospective cohort follow-up study of all children with a CHD born to women in the Greater Paris area (Paris and its surrounding suburbs) between 2005 and 2008 [18].

All cases (live births, pregnancy terminations, fetal deaths) diagnosed in the prenatal period or up to one year of age in the birth cohorts between May 1st 2005 and April 31st 2008 were eligible for inclusion. Multiple sources of data including all maternity units, pediatric cardiology and cardiac surgery centers, fetal and neonatal pathology departments, neonatal and pediatric intensive units, infant units and outpatient clinics in the catchment area as well as a neighboring tertiary care center were regularly consulted to attain completeness of care registrations.

**Fig. 1:**
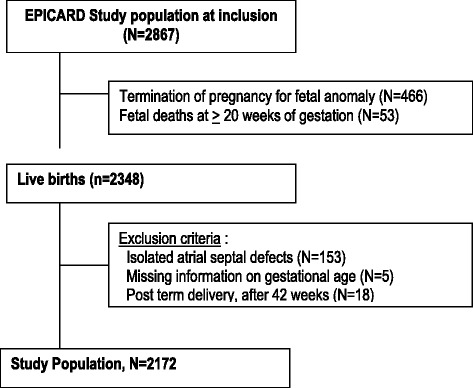
Flow chart – Study Population

The total number of births (live births + stillbirths) in the study population base was 317,538, which included 314,022 live births. The total number of cases (live births, terminations of pregnancy for fetal anomaly and fetal deaths at >20 weeks of gestation) included in the EPICARD cohort was 2867 (Fig. 1). After excluding terminations of pregnancy for fetal anomaly (*N* = 466) and fetal deaths (*N* = 53), our initial study population comprised 2348 live births. Five (0.2%) cases had missing information on gestational age and were excluded from the analyses. We also excluded isolated atrial septal defects (*N* = 153, 6.5%) (ASD) in order to minimize any ascertainment bias. Preterm infant are more likely to undergo echocardiography resulting in detection of minor isolated ASD that may otherwise go undiagnosed. In addition, isolated minor ASD may be difficult to distinguish from patent foramen ovale. Finally, we excluded post term delivery (i.e. delivery after 42 weeks). After these exclusions, there were 2172 newborns in our final study population.

Cardiac anomalies associated with a known chromosomal anomaly accounted for 6.2% (*N* = 134), and those with anomalies of other systems, including genetic syndromes, 15.5% (*N* = 337) of newborns with CHD.

### Statistical analysis

We compared the risk of infant mortality for newborns with preterm (gestational age < 37 weeks) vs. term (37–41 weeks) births for (i) all cases of CHD, (ii) “isolated” CHD (all cases excluding those associated with chromosomal anomalies or anomalies of other systems, including genetic syndromes; (iii) “isolated major” CHD (“isolated” CHD excluding VSD, as the latter are most frequently benign CHD that do not require any intervention).

We conducted separate analyses for timing of infant mortality for the following periods: early neonatal (< 1 week), late neonatal (7–27 days), early post-neonatal (28 days to three months) and late post-neonatal (three month to one year of age).

We calculated proportions with 95% binomial exact confidence intervals. We used poisson regression to compare the risk of death for preterm vs. term births.

In addition, for “isolated” and “isolated major” CHD, we used Kaplan-Meier survival curves to represent the risk the death over time for newborns in the following gestational age groups: 28–31 weeks, 32–34 weeks, 35–36 weeks and 37–41 weeks. Newborns with gestational age < 28 weeks were excluded as the sample size (*N* = 10) was not sufficient to examine this group separately. We used the log-rank test to determine the statistical significance of differences in the survival curves.

We used the Cox proportional hazard model to compare the risk (hazard) of death for the four gestational age groups noted above after taking into account the effects of the following potentially confounding variables: maternal age, occupation, geographic origin, diabetes mellitus, intra-uterine growth restriction (IUGR, <10th percentile) and multiple births. We tested the assumption of proportional hazards in the Cox model and found no statistically significant evidence of violation of this assumption in the Cox model. The R 2.13.2 software (R Development Core Team (2009), http://cran.r-project.org/) was used for data analysis.

## Results

### Study population

Table 1 shows the characteristics of the study population for preterm (< 37 weeks) and term (37–41 weeks) newborns with CHD. Overall, 13.6% (95% CI 12.2–15.1) of newborns were preterm. Median gestational age at birth was 39 weeks for term infants, and 35 weeks for preterm infants.

**Table 1:** Maternal and fetal characteristics of the study populationn

		Term (*N* = 1876)		Preterm (*N* = 296)		p
		n	%	n	%	
Characteristics						
Fetal	Gestational age: median(range)	39 (37–41)		35 (24–36)		
	Birthweight: median(range)	3240 (1380–5550)		2080 (500–4770)		<0.001
	IUGR (< 10th percentile)	223	11.9	67	22.6	<0.001
	Multiple pregnancy	45	2.4	142	48.0	0.9
	Male	894	47.7			
	Associated anomalies					
	chromosomal	95	5.1	39	13.2	<0.001
	other systems	259	13.8	74	25	<0.001
Maternal	Age in years: mean	31.1		32.3		0.002
	< 30	710	38.1	89	30.3	<0.001
	30–34	652	35.0	103	35.0	
	35–39	376	20.2	63	21.4	
	≥ 40	124	6.7	39	13.3	
	Nulliparous	898	48.3	137	46.4	0.5
	Geographic origin					
	France	930	49.9	116	39.2	<0.001
	North african	362	19.4	65	22.0	
	African	228	12.2	57	19.2	
	Other	346	18.5	58	19.6	
	Maternal occupation					
	professional	444	25.2	49	17.6	0.01
	intermediate	354	20	46	16.6	
	administrative/public service	203	11.5	39	14.0	
	other	285	16.2	50	18.0	
	none	479	27	94	33.8	
	Diabete mellitus	82	4.4	21	7.1	0.04
	Prenatal diagnosis of CHD	375	20.0	73	24.7	0.06

Mothers of preterm newborns were on average older (31.1 vs. 32.3 years *p* < 0.001), and more likely to be unemployed or from African origin. Preterm newborns were more likely to have associated chromosomal (13.2% vs. 5.1% for full term newborns) or other anomalies (24.3% vs. 14.1%) and IUGR (22.6% vs. 11.9%). Multiple pregnancies accounted for 17.9% of preterm vs. 2.4% of term newborns (all *p*-values <0.05). The proportion of maternal diabetes was higher (7.1% vs. 4.4%) and that of prenatal diagnosis lower (20.0% vs. 24.7%) for preterm vs. term newborns; however, these differences did not reach statistical significance (*p*-values 0.08 and 0.07, respectively).

The overall infant mortality rate was 6.5%. Preterm newborns had a 3.8-fold higher risk of infant death (17.9%) than term newborns (4.7%), RR 3.8, 95%CI 2.7–5.2 (Table 2). The higher risk of mortality for preterm newborns appeared to be greater in the neonatal (RR 4.3, 95% CI 2.9–6.6) than in the post-neonatal (RR 3.0, 95% CI 1.7–5.2) period. However, the confidence intervals were relatively wide and overlapping.

**Table 2:** Risk on infant death in term ans preterm newborns: all cases, isolated CHD^a^ and isolated majour CHD^b^

		Term		Preterm			
		%	95% CI	%	95% CI	RR	95% CI
All cases		*N* = 1876		*N* = 296			
Neonatal mortality	early <7 days	1.5	1.0–2.1	6.1	3.6–9.4	4.1	2.3–7.3
	7.3late 7-28d	1.2	0.8–1.8	5.7	3.4–9.0	4.7	2.5–8.7
	Total	2.7	2.0–3.6	11.8	8.4–16.1	4.3	2.9–6.6
Post neonatal mortality	28d-3 months	0.9	0.5–1.4	2.4	1.0–4.8	2.8	1.2–6.7
	3 m-1y	1.2	0.7–1.8	3.7	1.9–6.6	3.2	1.6–6.5
	Total	2.1	1.4–2.8	6.1	3.6–9.4	3.0	1.7–5.2
Infant mortality	<1 year	4.8	3.8–5.8	17.9	13.7–22.8	3.8	2.7–5.2
Isolated CHD^a^		*N* = 1551		*N* = 200			
Neonatal mortality	early <7 days	1.0	0.5–1.6	3.0	1.1–6.4	3.1	1.2–7.9
	7.9late 7-28d	0.8	0.5–1.4	2.5	0.8–5.7	3	1.1–8.3
	Total	1.8	1.2–2.6	5.5	2.8–9.6	3.0	1.5–6.0
Post neonatal mortality	28d-3 months	0.5	0.2–1.0	1.5	0.3–4.3	2.9	0.8–10.8
	3 m-1y	0.8	0.4–1.4	1.0	0.1–3.6	1.3	0.3–5.7
	Total	1.3	0.8–2.0	2.5	0.8–5.7	1.9	0.7–5.1
Infant mortality	<1 year	3.1	2.2–4.0	8	4.6–12.8	2.6	1.5–4.5
Isolated major CHD^b^		*N* = 542		*N* = 114			
Neonatal mortality	early <7 days	2.8	1.6–4.5	5.3	2.0–11.1	1.9	0.8–4.8
	late 7-28d	2.4	1.3–4.1	4.4	1.4–9.9	1.8	0.7–5.0
	Total	5.2	3.5–7.4	9.7	4.9–16.6	1.9	1.0–3.6
Post neonatal mortality	28d-3 months	1.5	0.6–2.9	2.6	0.6–7.5	1.8	0.5–6.6
	3 m-1y	2	1.0–3.6	1.8	0.2–6.2	0.9	0.2–3.8
	Total	3.5	2.1–5.4	4.4	1.4–9.9	1.3	0.5–3.3
Infant mortality	<1 year	8.7	6.4–11.4	14.1	8.2–21.8	1.6	1.0–2.8

^a^Isolated CHD: excluding cases with chromosomal or other anomalies

^b^Isolated major CHD: excluding cases with chromosomal or other anomalies and ventricular septal defects (VSD)

After excluding chromosomal and other anomalies, risk of infant mortality for preterm newborns with “isolated” CHD was 2.6-fold higher than term newborns (RR 2.6, 95% CI, 1.5–4.5). For “isolated major” CHD (i.e., CHD excluding cases associated with chromosomal or other anomalies and VSD), risk of infant mortality associated with PTB was 1.6-fold higher than for term newborns (RR 1.6; 95%CI 1.0–2.8) (Table 2).

### Survival analysis

**Fig. 2:**
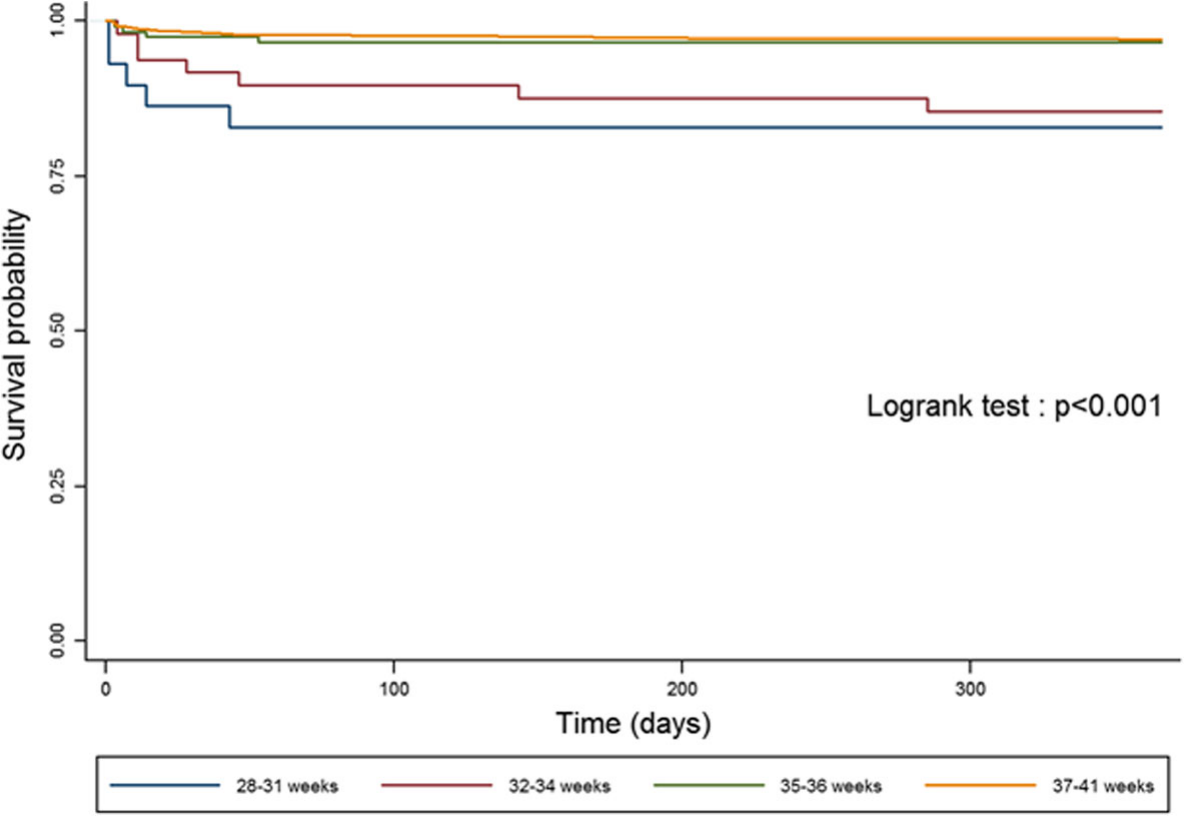
Gestational-age specific Kaplan-Meier survival curves for infants with isolated CHD

**Fig. 3:**
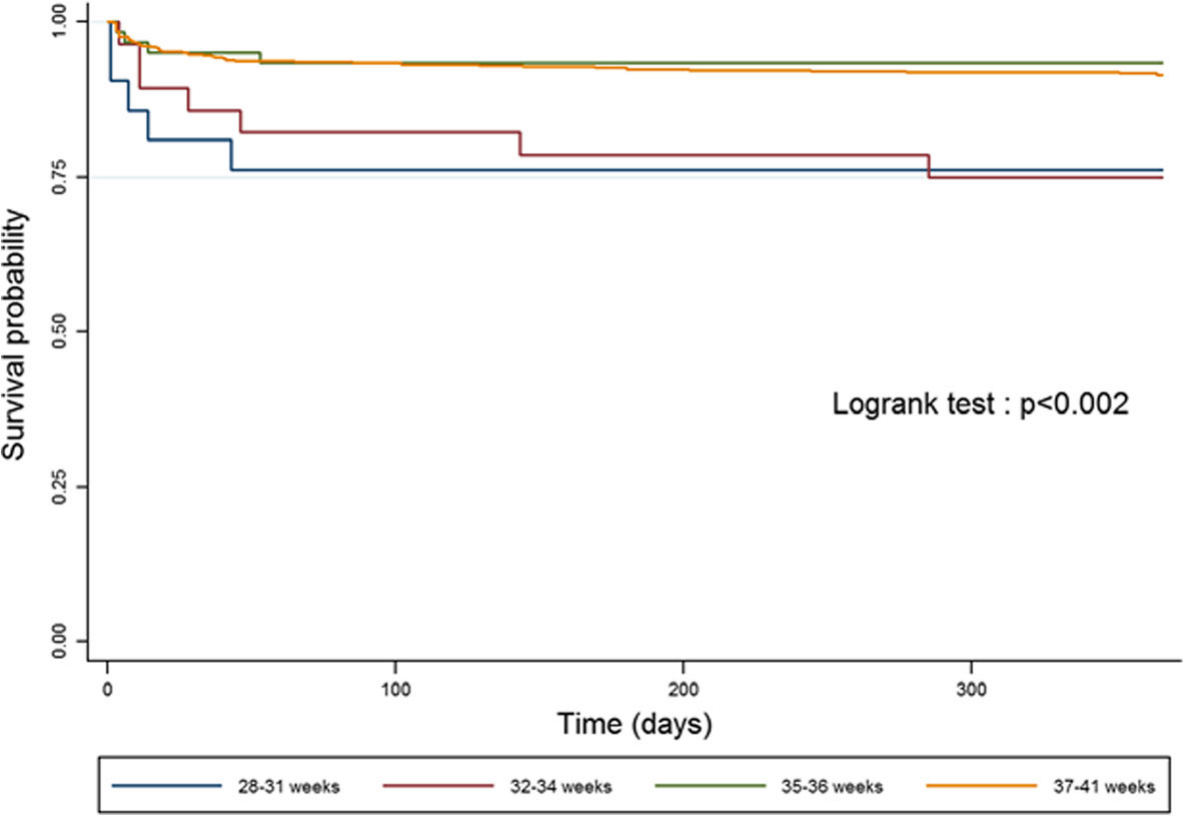
Gestational-age specific Kaplan-Meier survival curves for infants with isolated *major* CHD

Figure 2 shows the survival curves for four gestational age groups: 28–31 weeks, 32–34 weeks, 35–36 weeks and 37–41 weeks for newborns with isolated CHD. Newborns with gestational age less than 35 weeks had significantly lower survival rates than those born at term whereas the survival curves for newborns 35–36 weeks were very similar to those born at term. Most of the differences across the survival curves were due to early deaths in the neonatal and early post-neonatal period. Differences in survival curves (Fig. 3) for isolated major CHD (isolated CHD, excluding VSD) paralleled those noted above; however the “baseline” risk of mortality was higher for all gestational age groups after exclusion of VSD.

Table 3 shows the results of the Cox proportional hazards models for estimating the hazard ratios of mortality across the four gestational age groups after taking into account the effects of potentially confounding factors including maternal age, occupation, geographic origin, diabetes mellitus, intra-uterine growth restriction (IUGR, <10th percentile) and multiple births. The adjusted hazard of mortality for children with isolated CHD was 4.0 (HR 4.0, 95%CI 1.5–10.5) and 5.4 (HR 5.4, 95%CI 2.1–13.9) higher for newborns at 28–31 weeks and 32–34 weeks of gestational age, respectively, as compared with term newborns. The hazard ratio for newborns at 35–36 weeks was not statistically significant (HR 0.9 95%CI 0.3–2.7). The adjusted hazard ratios associated with low gestational age groups for newborns with isolated major CHD were 2.1 (95% CI 0.8–5.4) and 3.1 (95% CI 1.2–8.1) for newborns at 28–31 and 32–34 weeks, respectively. There was no evidence of a difference in the hazard of death between newborns at 35–36 weeks vs. term newborns (HR 0.6, 95% CI, 0.2–1.9) for newborns with “isolated major” CHD.

**Table 3:** Cox proportional hazard models of the impact of preterm birth on the risk of infant death

		HR	95% CI	p^c^	HRa^d^	95%CI	p^c^
Isolated CHD^a^							
Gestational age (weeks)	28–31	6.5	2.6–16.3	<0.001	4.0	1.5–10.4	<0.001
	32–34	4.9	2.2–10.9		5.4	2.1–13.9	
	35–36	1.1	0.4–3.1		0.9	0.3–2.7	
Isolated major CHD^b^							
Gestational age (weeks)	28–31	3.3	1.3–8.4	0.003	2.1	0.8–5.4	0.045
	32–34	3.1	1.4–6.8		3.1	1.2–8.0	
	35–36	0.8	0.3–2.1		0.6	0.2–1.9	

^a^Isolated CHD: excluding cases with chromosomal or other anomalies

^b^Isolated major CHD: excluding cases with chromosomal or other anomalies and ventricular septal defects (VSD)

^c^Likelihood ratio test

^d^adjusted for maternal age, occupation, geographic origin, diabetes, intra-uterine growth restriction (IUGR) and multiple pregnancy

## Discussion

Using population-based data on 2172 newborns with CHD, we found that the risk of infant mortality was about four-fold higher for preterm vs. term infants with CHD. The relative risk associated with PTB was lower (RR ~ 2.6) after cases with associated chromosomal or other anomalies were excluded and lowest in case of “isolated major” CHD (“isolated” CHD, VSD-excluded) (RR ~ 1.6). Survival analysis estimates suggested that the higher risk of mortality associated with PTB was limited to newborns with gestational age < 35 weeks and was disproportionately due to early deaths in the neonatal and early post-neonatal period. The higher risk of mortality associated with preterm births <35 weeks remained statistically significant and clinically important after exclusion of other anomalies and adjustment for potentially confounding factors.

Our estimate for the overall risk of infant mortality for preterm newborns with CHD (17.9%) are comparable to those reported by Tanner et al. in a population-based study in the British population [5]. However, in that study the authors did not examine the risk of mortality for preterm newborns in detail. In particular, the timing of mortality, the role of associated anomalies and the impact of potentially confounding factors were not analyzed.

Our estimate of the relative risk of infant mortality associated with preterm birth for newborns with CHD (RR ~ 3.8) was lower than the relative risk of mortality associated with preterm birth in the general population for several European countries and the United States; where the relative risks of infant mortality associated with preterm birth were found to be consistently greater than ten [19, 20]. This lower RR of preterm birth in newborns with CHD is of course not due to any, as it were, protective effect of CHD on the risk of mortality associated with preterm birth. Instead, this is most likely due to the fact that in newborns with CHD, particularly those with severe CHD, preterm birth may play a smaller role as a prognostic factor since the CHD pathology, in cases of moderately severe and more so severe CHD (e.g., functionally univentricular heart) can be a more important prognostic factor than the gestational age of the newborn. Indeed, we found that the relative risk associated with PTB was lower when cases of VSD, which for the most part represent benign lesions that do not require intervention, were excluded. This suggests in turn that as the severity of CHD increases, the effect of PTB, *relatively* speaking, decreases.

In addition, the effect of PTB on the risk of mortality of newborns with CHD may be in part due to differences in the spectrum of CHD associated with preterm vs. term births. A previous study [6] found that in general categories of CHD that were associated with a higher risk of PTB were also more likely to represent more severe cases of CHD.

Our study has certain limits. Even though our study population included more than 2000 cases of newborns with CHD, we were not able to examine the risk of mortality separately for extremely (< 28 weeks) PTB due to our limited sample size for this gestational age group. In addition, our study was not designed to assess the impact of different management strategies (e.g., timing of surgery [8, 12, 21–24]) for preterm vs. term newborns with CHD. We also could not examine in any detail the extent to which differences in mortality between preterm vs. term newborns with CHD may be due to differences in the spectrum of CHD or the extent to which the effect of PTB may be modified by different type of CHD.

It should be noted that while we took into account the potentially confounding effects of certain socioeconomic factors, the scope of our study was much more limited than a comprehensive analysis of the many factors associated with PTB and CHD, and in particular the potential role of socioeconomic factors such as education, income and place of residence, would require. Future studies should examine in particular the extent to which any effects of socioeconomic factors on the risk of infant mortality may be due to (mediated by) preterm birth, which is known to vary across socioeconomic groups.

## Conclusion

We found that overall preterm newborns with CHD, particularly those who were born at <35 weeks of gestational age, had a substantially higher risk of mortality than those born at term (37–41). This higher risk decreased but remained statistically significant after exclusion of associated anomalies and adjustment for potentially confounding factors, including maternal sociodemographic factors, multiple pregnancies and IUGR. The *relative risk* of mortality associated with PTB was substantially lower for newborns with CHD than the comparable estimates in the general population. In terms of absolute risk differences, however, the situation was different. This is so because a given relative risk of mortality associated with preterm birth for infants with CHD “translates” into a higher absolute risk difference since the baseline risk of mortality for full-term infants with CHD is higher than full-term infants in the general population.

Future studies should examine the extent to which the higher risk of mortality associated with PTB may vary across the spectrum of CHD and assess the role of alternative clinical management strategies (e.g., age at surgery) on survival and long-term neuro-developmental outcomes of preterm newborns with CHD.

## Abbreviations

ASD: Atrial septal defect
CHD: Congenital heart defect
CI: Confidence interval
EPICARD: Etude EPIdémiologique sur le devenir des enfants porteurs de CARDiopathies congénitales
HR: Hazard ratio
IUGR: Intrauterine growth restriction
OR: Odds ratio
PTB: Preterm birth
VSD: Ventricular Septal Defect
